# Phylogenomics and population genomics of SARS-CoV-2 in Mexico during the pre-vaccination stage reveals variants of interest B.1.1.28.4 and B.1.1.222 or B.1.1.519 and the nucleocapsid mutation S194L associated with symptoms

**DOI:** 10.1099/mgen.0.000684

**Published:** 2021-11-30

**Authors:** Francisco Barona-Gómez, Luis Delaye, Erik Díaz-Valenzuela, Fabien Plisson, Arely Cruz-Pérez, Mauricio Díaz-Sánchez, Christian A. García-Sepúlveda, Alejandro Sanchez-Flores, Rafael Pérez-Abreu, Francisco J. Valencia-Valdespino, Natali Vega-Magaña, José Francisco Muñoz-Valle, Octavio Patricio García-González, Sofía Bernal-Silva, Andreu Comas-García, Angélica Cibrián-Jaramillo

**Affiliations:** ^1^​ Unidad de Genómica Avanzada (Langebio), Cinvestav-IPN, Irapuato, Guanajuato, Mexico; ^2^​ Departamento de Ingeniería Genética, Unidad Irapuato, Cinvestav-IPN, Irapuato, Guanajuato, México; ^3^​ Conacyt – Unidad de Genómica Avanzada (Langebio), Cinvestav-IPN, Irapuato, Guanajuato, México; ^4^​ Molecular Biology Research & Development Department, GrupoT, Irapuato, Guanajuato, México; ^5^​ Facultad de Medicina, Universidad Autónoma de San Luis Potosí, San Luis, San Luis Potosí, México; ^6^​ Instituto de Biotecnología, Universidad Nacional Autónoma de México, Cuernavaca, Morelos, México; ^7^​ Centro de Investigación en Matemáticas AC (Cimat), Sede Aguascalientes, Aguascalientes, México; ^8^​ Prothesia, Monterrey, Nuevo Leon, México; ^9^​ Laboratory for the Diagnosis of Emerging and Reemerging Diseases (LaDEER), University Center for Health Sciences, University of Guadalajara, Guadalajara, Jalisco, México; ^10^​ Institute for Research in Biomedical Sciences, University Center for Health Sciences, University of Guadalajara, Guadalajara, Jalisco, México; ^11^​ Centro de Investigación en Ciencias de la Salud y Biomedicina, Universidad Autónoma de San Luis Potosí, San Luis, San Luis Potosí, México

**Keywords:** SARS-CoV-2, population genomics, nucleocapsid, Mexico, asymptomatic, variants

## Abstract

Understanding the evolution of the SARS-CoV-2 virus in various regions of the world during the Covid-19 pandemic is essential to help mitigate the effects of this devastating disease. We describe the phylogenomic and population genetic patterns of the virus in Mexico during the pre-vaccination stage, including asymptomatic carriers. A real-time quantitative PCR screening and phylogenomic reconstructions directed at sequence/structure analysis of the spike glycoprotein revealed mutation of concern E484K in genomes from central Mexico, in addition to the nationwide prevalence of the imported variant 20C/S:452R (B.1.427/9). Overall, the detected variants in Mexico show spike protein mutations in the N-terminal domain (i.e. R190M), in the receptor-binding motif (i.e. T478K, E484K), within the S1–S2 subdomains (i.e. P681R/H, T732A), and at the basis of the protein, V1176F, raising concerns about the lack of phenotypic and clinical data available for the variants of interest we postulate: 20B/478K.V1 (B.1.1.222 or B.1.1.519) and 20B/P.4 (B.1.1.28.4). Moreover, the population patterns of single nucleotide variants from symptomatic and asymptomatic carriers obtained with a self-sampling scheme confirmed the presence of several fixed variants, and differences in allelic frequencies among localities. We identified the mutation N:S194L of the nucleocapsid protein associated with symptomatic patients. Phylogenetically, this mutation is frequent in Mexican sub-clades. Our results highlight the dual and complementary role of spike and nucleocapsid proteins in adaptive evolution of SARS-CoV-2 to their hosts and provide a baseline for specific follow-up of mutations of concern during the vaccination stage.

## Data Summary

The Mexstrain-Nextstrain data used for this study can be found at: http://www.ira.cinvestav.mx/ncov.evol.mex.aspx.

The data generated and used in this study are provided as supplementary tables: Table S1. Self-sampling performance validation data generated in 58 volunteers. Table S2. Metadata of SARS-CoV-2 genomes generated and investigated. Table S3. Complete dataset of the population genomics analysis of SARS-CoV-2.

**Impact Statement**

Following self-sampling, screening of mutations of concern, and a combined phylogenomic and population genetics pipeline of the SARS-CoV-2 virus, we reveal the appearance of specific mutations of concern in the spike protein that define the postulated variants of interest P.4 (B.1.1.28.4) and 20B/478K.V1 (B.1.1.222, leading to B.1.1.519), and in the nucleocapsid protein, N:S194L, in Mexico during the pre-vaccination stage. The mutation S194L in the nucleocapsid was found to associate with symptomatic patients versus asymptomatic carriers in the population investigated. Our research can aid epidemiological genomics efforts during the vaccination stage in Mexico by providing a combined analytical platform and information about variants within different genetic lineages with the potential to evolve into further variants of interest or of concern.

## Introduction

Mutation D614G of the SARS-CoV-2 spike protein was the first mutation implicated in increased transmission and a more efficient viral replication in human cells [1]. It emerged as a consequence of natural selection in the S1 region of the spike protein as the virus colonized human populations outside of Asia to Europe and to the rest of the world [2]. Shortly after the appearance of D614G, other mutations in the Receptor Binding Domain (RBD) of the spike protein appeared, including but not limited to K417N, L452R, E484K and N501Y. Their increasingly high frequency in recent months, their potential for increased transmission coupled with immunological escape [3–7], and the probable increased mortality [8] have alerted the medical and scientific community to follow emerging and prevalent variants of interest (VOI) and variants of concern (VOC) [9, 10]. VOI and VOC are both defined as highly prevalent versions of the virus with several mutations of concern, which may provide an increased fitness to the virus due to phenotypic changes that result in an increased threat to human health [11]. VOI are preceded by alerts for further monitoring by the World Health Organization (WHO) and require experimental evidence of the suspected phenotypic dangerous trait to become VOC.

Emerging variants can be detected by their phylogenetic position provided by the Nextstrain [12] and Pangolin platforms, the latter being the primary source of classification and nomenclature of SARS-CoV-2 [13]. Defining mutations included in VOI and VOC can also co-occur with other mutations, both within the variable N-terminal domain (NTD) region and/or the flexible RBD of the spike protein or throughout the SARS-CoV-2 genome. A collection of functionally meaningful non-synonymous point-mutations that may arise independently several times leads to the so-called Covid-19 constellations (https://github.com/cov-lineages/constellations). Such co-occurring mutations, including indels, are expected given the virus’ current evolutionary trajectory. Of the 16 emerging lineages identified during the summer of 2020, four evolved early in 2021 into lineages with VOC bearing signature shared mutations: 20I/501Y.V1 (UK, B.1.1.7, alpha), 20h/501Y.V2 (Republic of South Africa, B.1.351, beta) and 20J/501Y.V3 (Brazil, P.1 or B.1.1.28.1, gamma); and 20C/S:452R (California, USA, B.1.427/9, epsilon). Together with the P.1 closely related VOI P.2 (B.1.1.28.2, zeta) these VOC/VOI often share mutations E484K and/or N501Y. Mutations defining VOC, e.g. E484K, are increasingly common worldwide as they are imported from their regions of origin into naive populations, but also as independent evolution converges to the same point mutations, such as in the recently reported VOI 20A/S.484K and 20C/S.484K (B.1.525/526, eta/iota [14, 15], amongst others.

These viral evolutionary emerging scenarios are of concern for their potential to extend the duration of the pandemic, as these lineages emerge and spread in unvaccinated populations concomitantly to vaccinated ones. The convergence of K417N, E484K and N501Y in different geographical regions suggest that during the ‘pre-vaccination stage’ of the pandemic, similar selection pressures took place to increase the fitness of SARS-CoV-2 [16]. For instance, 20J/501Y.V3 or P.1 evolved in parallel with the 20B/S.484K or P.2 variant in Brazil, and they share the spike protein mutations E484K and V1176F, but not N501Y. Likewise, although geographically unrelated, it can be argued that the places of origin of VOC 20I/501Y.V1, 20h/501Y.V2, 20J/501Y.V3 and 20C/S:452R have in common large populations with worrying recorded epidemiological statistics (deaths, cases, R0, positivity, etc.). Cases where natural or unintended immunological pressures, such as the use of convalescent plasma [17] or high prevalence of the disease leading to a high immunological burden [11], can help us understand what to expect during the vaccination stage of the pandemic, but further population genomics and epidemiological studies are needed.

The finer scale population genetic patterns of the virus as it adapts to local conditions is a recent focus of research. SARS-CoV-2 has a high-fidelity transcription and replication process [18] compared to other ssRNA viruses, resulting in generally lower genetic diversity [19]. Yet the large SARS-CoV genomes allow efficient exploration of the sequence space [20] so the array of possible variants during SARS-CoV-2’s trajectory as it adapts to our large populations and even crosses over to other species [21] is yet to be described in full. One of the few previous population-level studies in SARS-CoV-2 showed that haplotype diversity and allele frequencies in infection clusters are probably caused by natural selection [22]. Additionally, in early 2020 there were two significant lineages identified by population-level analyses [23]; in late 2020, there were six viral subtypes based on the estimation of increased nucleotide diversity [24, 25]; and in 2021, there were four sub-strains in the USA alone detected by population-level analyses [26].

Here, we provide a baseline analysis for the evolution of SARS-CoV-2 in Mexico between March 2020, when the first case of Covid-19 was registered in the country, and February 2021, when large-scale vaccination started. Based on such analysis, we postulate the occurrence of two VOI: P.4, which evolved from 20B/P.2, and 20B/478 K.V1 or B.1.1.222/B0.1.1.519, emerging within Mexico. We use publicly available genomic data for Mexico, plus 85 genomes generated by us that include asymptomatic carriers identified using a novel self-sampling strategy developed to increase success rate in sampling these individuals without uncertainty and reduced risk. Based on this, we identified mutation N:S194L of the nucleocapsid to be potentially associated with the development of symptoms. Thus, two specific SARS-CoV-2 scenarios, the prevalence of mutations of concern and population-level viral allelic variation, are reported, while providing an ad hoc bioinformatics pipeline for real-time analysis of SARS-CoV-2 genomes in Mexico. Our report is timely as it coincides with a national epidemiological genomics programme that aims at providing a thousand genomes per month from hospital samples throughout the country; and it is of interest as the current vaccination programme in many countries, including Mexico, involves diverse vaccine technologies.

## Methods

### Self-sampling for identification of asymptomatic SARS-CoV-2 carriers and sample preparation

We sampled asymptomatic SARS-CoV-2 carriers from private organizations represented by a Human Resources (HR) in the City of Irapuato, State of Guanajuato. The HR contact person would report on suspected cases of Covid-19 with symptoms, which were sent to the clinic for an assessment and diagnosis by healthcare professionals. At this point, all contacts of the suspected Covid-19 patient were identified through contact tracing, including their family members, and provided with a self-sampling kit with instructions for collection of nasopharyngeal swabs (Fig. S1, available in the online version of this article). Validation in 58 volunteers was done by self-sampling and assisted sampling by a qualified healthcare professional, followed by quantification of nucleic acids with a Qubit Flex Fluorometer (Thermo Fisher), and by quantitative PCR (qPCR) amplification of the human marker RNAse P as control (Table S1).

Additive manufacturing technology became an essential player as supply shortages affected swab and viral transport media (VTM) production globally. Our group used 3D printing for emergency manufacturing devices, including face shields and swabs. For this, we partnered with Prothesia (https://www.prothesia.com/covid19), a Mexico-based company dedicated to creating software algorithms to manufacture custom orthotic devices. FDA-cleared 3D-printed nasopharyngeal swabs designed by USF-Northwell and manufactured by Prothesia were used for self-sampling. Manufacturing was done using methacrylate monomers, urethane dimethacrylate and a photoinitiator as reagents, with stereolithography technology on Formlabs printers, curing and washing stations. The tip of the swab features a biomimetic design based on the ‘cattail’ (Typha) plant, and it has a rounded nose to maximize comfort and lateral alternating nubs to maximize surface area for sample collection.

Samples were transported and saved in 700 µl of VTM (Atila Biosystems) and/or DNA/RNA Shield (Zymo Research). The DNA was saved for future investigations. This provided enough material for two extractions of RNA, each of 300 µl, done with a chemagic Viral DNA/RNA 300 Kit H96 from PerkinElmer, Quick-RNA Viral Kit (Zymo Research) or QIAmp Viral RNA Mini Kit (Qiagen); and one extraction of DNA of the entire 700 µl, done with a ZymoBiomics DNA Kit. Around 31.5 ng µl^−1^ of RNA and 73.3 ng µl^−1^ of DNA were obtained per extraction in a total volume of ˜50 µl. The RNA material was used for SARS-CoV-2 diagnostics using suitable thermocyclers for real-time (RT)-qPCR, with kits validated by InDRE and provided by Genes2Life, to the academic diagnostics laboratories of Guanajuato (Wov19) and Jalisco (Decov triplex). Detection of San Luis Potosí SARS-CoV2-positive samples was done with the GeneFinder COVID-19 PLUS RealAmp Kit.

### Identification of mutations of concern

Covid-19 variants, whether VOI or VOC (or their preceding ‘Alerts for Further Monitoring’ by the WHO), are defined as versions of the virus with a collection of mutations. From this, non-synonymous and functionally meaningful point-mutations present in VOI or VOC are used here to define ‘mutations of concern’, such as E484K. Screening for E484K and N501Y mutations was done by RT-qPCR, using module 2 of the MUT-SARS-CoV-2 kit (Genes2Life SAPI de CV) simultaneously in the same reaction using RNA previously identified as positive for SARS-CoV-2, as previously described [27]. The reactions were carried out using a QuantStudio 5 RT System thermal cycler from Applied Biosystems. This assay uses four fluorescent probes that specifically hybridize with the target sequences to discriminate each base/mutation. Thus, detection and discrimination are carried out in a fourplex assay, the channels being for the FAM and HEX dyes dedicated to detection of wildtype background; and the Cal Fluor Red 610 and Quasar 670 dye channels used for detection of the E484K and N501Y mutations, respectively. A total of 1560 RNA samples from the States of San Luis Potosí (170), Jalisco (1330) and Mexico City (60), previously identified as positive for SARS-CoV-2, were screened.

### Genome assembly and sequencing of SARS-CoV-2

Two different approaches were used to produce sequencing libraries. A total of 78 cDNA samples from Guanajuato (45) and San Luis Potosi (33) were prepared using the Illumina/IDbyDNA Respiratory Pathogen ID/AMR Enrichment Kit following the vendor’s protocol for library preparation. Libraries were prepared with Illumina RNA Prep with Enrichment (Illumina; Catalogue no. 20040536) and IDT for Illumina DNA/RNA UD Indexes (Illumina; Catalogue no. 20027213). Illumina RNA Prep with Enrichment consists of an On-Bead Tagmentation followed by a single hybridization step to generate enriched DNA and RNA libraries. After amplification, libraries were enriched as 3-plex reactions using the Illumina Respiratory Pathogen ID/AMR Panel to detect several viral (RNA) and bacterial (DNA) pathogens, including SARS-CoV-2, via probe capture. The DNA bacterial data will be reported elsewhere. Libraries were sequenced on an Illumina NextSeq 500 platform using a configuration for 75 bp paired-end reads. A further 24 sequencing libraries from Jalisco (nine) and San Luis Potosí (15) were constructed using the QIAGEN library preparation kit QIAseq_SARSCov2_Primer_Panel. Shotgun sequencing was done in an Illumina MiSeq 2×150 v3 platform. SARS-CoV-2 genome sequences were generated by mapping reads to the NC_045512.2 reference genome using the Explify pipeline at the Illumina BaseSpace hub site or with the Illumina DRAGEN Bio-IT Platform. Of the 102 genomic libraries generated, only 35 reached GISAID quality for phylogenomic analysis and 85 were suitable for population genomics. Details on these genome sequences are provided in Table S2.

### Phylogenomics of SARS-CoV-2 mutations of concern

All complete and high-coverage SARS-CoV-2 genome sequences from Mexico (from samples obtained up to February 2021) were downloaded from GISAID in March 2021 (https://www.gisaid.org/): 1554 genomes in total. We also downloaded from GISAID all genome sequences represented in Nextstrain SARS-CoV-2 global analysis (https://nextstrain.org/ncov/global, date: March 2021). We joined the above set of sequences (and their associated metadata) into a single set for phylogenomic analyses by using in-house Perl scripts (https://github.com/luisdelaye/Mexstrain), giving place to our ad hoc Mexstrain platform (http://www.ira.cinvestav.mx/ncov.evol.mex.aspx) for phylogenomic analyses using a local version of Nextstrain [12]. We used a ‘global’ sampling scheme grouping 10 sequences per ‘country year month’ and modified the defaults/include.txt file to include all Mexican sequences. For the rest of the parameters, we used those provided by the ncov Nextstrain build. All configuration files are available upon request. The obtained phylogenies were used to manually investigate mutations of concern according to Pangolin nomenclature [13].

### Incidences and relationships between SARS-CoV-2 mutations of concern

We focused on the gene coding for the spike protein. All 1554 Mexican SARS-CoV-2 sequences were compared against MN908947 as a reference used by Nextstrain (the sequence MN908947 used by Nextstrain version 3.0.3 is identical to NC_045512.2). We identified 337 combinations of mutations and 315 unique mutations from 1552 sequences using in-house Perl and Python scripts (two sequences were filtered out because of quality issues). To identify homologous positions between Mexican and reference sequences, we used the multiple sequence alignment generated by our local Nextstrain installation. We transformed the output file to study the incidence for 315 mutations grouped in 11 clades, and we studied their covariances between one another using in-house scripts with the R packages tidyverse [28] and circlize [29], and Python modules NumPy [30], Pandas [31], matplotlib [32], seaborn [33] - R version 4.0.4 [34], R Studio 1.4.1106 [35], Python 3.8 [36] and JupyterNotebook 6.1.4 [37] (https://github.com/plissonf/Phylogenomics_SARS-CoV-2_Mexico).

### Population genomics of SARS-CoV-2

Nucleotide variants and small indels allow the assessment of allele frequencies at the population level and the intra-host co-occurrence of ancestral (known Wuhan reference) and derived (alternative) alleles that can inform the appearance of new variants and their evolution. We designed a mapping-based approach to call single nucleotide variants (SNVs) and indels directly from sequencing reads rather than assembled genomes. Due to the possibility of having SNPs due to RNA modification rather than RNA replication errors, we did not separate allele frequencies (i.e. A–G and C–T mismatches) that could potentially be due to deamination events [38], so we refer to nucleotide variation at the population level as SNVs more generally. Also, most Cycle Treshold (CT) values ranged between 28 and 30, suggesting an intermediate to high viral load in our samples [39], reducing the possibility of biases in allele frequencies due to lower viral loads [40]. Raw reads from a total of 102 paired-end libraries were processed by first removing adapters, low-quality bases and short reads using the fastP program [41] using default parameters. The reads were mapped to the NC_045512.2 version of the SARS-CoV-2 reference genome using BWA [42] with default parameters. Sam alignments were converted to bam files and sorted using samtools [43]. Bam files were used to call variants (SNVs+indels) via the freebayes program [44]. VCF files were then concatenated and parsed to retain only biallelic SNVs supported by at least 20 sequencing reads and alignment qualities above 30 using in-house R scripts. Polymorphic site frequencies were estimated based on 85 samples that passed our filters. Two samples were removed because they contained unique variants segregating in <5 % of the total samples. Locality-wise polymorphic site frequencies were then estimated by pondering the 43 GTO sample, 31 SLP samples and nine JAL samples. The genome-wide site frequency spectrum was then assessed per locality employing a probability density function. In addition to exploring the differences in polymorphic site frequencies among localities, we used ANGSD [45] to construct a genetic covariance matrix between samples using genotype likelihoods directly from the bam files, and used this matrix for a principal components analysis using the prcomp function of R. To explore the prevalence of SNVs within hosts (intra-host variation), we identified and estimated the ‘allelic imbalance’, defined as the proportion of total reads that support either the ancestral or the derived allele, per polymorphic biallelic site. The discrete statistical association of the expression of symptoms and the presence of specific mutations was assessed by means of an exact binomial test for sites with at least 15 samples. To analyse the effect of the intra-host allelic imbalance on the expression of symptoms, we performed a Wilcoxon test collapsing all polymorphic sites within groups. A one-way ANOVA was then employed to test the individual effects of allelic imbalance at each polymorphic site to the expression of symptoms. Data visualization and statistical analysis were performed using the tidyverse [28], ggridges [46] and rstatix [47] R libraries.

### Positions of mutations of concern in the spike protein of SARS-CoV-2

We extracted the electron microscopy structure (7A94 [48], of the trimeric spike (S) glycoprotein of SARS-CoV-2 bound to one Angiotensin-Converting Enzyme-2 (ACE2). We depicted different domains and subdomains of S in various colours and textures (cartoon, surfaces) using MacPyMOL v.1.7 (Schrodinger LLC, https://pymol.org/). We rendered 3D representations with the program ray before proceeding to screen captures. We marked the important mutations S477N, T478K, E484K, D614G, P681H/R and T732A.

## Results

### Sampling of asymptomatic SARS-CoV-2 carriers

It was relatively straightforward to have access to samples from Covid-19 patients approaching the diagnostics laboratory or the hospital (samples from San Luis Potosi and Jalisco States), but this was not the case for asymptomatic carriers of the SARS-CoV-2 virus from Guanajuato. We solved this problem by tracing back contacts of patients with symptoms and by using a *de novo* self-sampling scheme to collect samples. We developed a highly efficient 3D polycarbonate swab that ensured capture of larger amounts of nucleic acids than obtained with traditional cotton swabs (or other materials), greatly reducing the risk of false negatives. The performance of the swab and self-sampling method was validated with 58 volunteers subject to nasopharyngeal sampling by a medical professional and self-sampling following simple instructions provided by us (see Methods and Fig. S1).

The self-sampling approach yielded a minimum amount of 20 ng µl^−1^ per extraction, carried out in duplicate. We obtained sufficient nucleic acids to perform several RT-qPCRs for separate diagnostic purposes or mutant screening, as well as the entire sequencing of their genomes. By following this approach, we also reduced the risk among members of the potential clusters investigated, and health professionals were under less viral exposure. For the State of Guanajuato, a total of 23 asymptomatic SARS-CoV-2 carriers, plus a total of 37 patients who eventually developed symptoms, could be identified after a screening of 484 individuals. Related metadata and genomic SARS-CoV-2 IDs are provided in Table S2.

### Targeted RT-qPCR identification of VOC in Mexico

We adopted an RT-qPCR approach to identify mutations of concern previously detected in VOC circulating worldwide, as previously described [27]. We screened for the spike protein mutations E484K, N501Y and 69–70 deletion. A total of 1560 SARS-CoV-2-positive samples from patients coming from Mexico City (60) and the States of San Luis Potosi (170) and Jalisco (1330) from the pre-vaccination stage were characterized. This allowed us to identify only one sample during January 2021 (frequency of 0.59%, 1/170) from the State of San Luis Potosi (Fig. S2b). The genome sequence of the associated SARS-CoV-2 virus was obtained by Mexican authorities (Mexico/SLP-InDRE_454/2021, GISAID EPI ISL 1219714), confirming the mutation E484K, and also, independently by us in this study, as the raw reads were required for our population genomics analysis. Likewise, following the same approach, a total of nine independent samples containing the E484K mutant could be detected in samples from the State of Jalisco (Fig. S2a). The genome sequences of three of these samples were generated by Mexican authorities (Mexico/JAL-InDRE_371, 372, 373/2021, GISAID EPI ISL 1093145, 1093146, 1093147), and us, including the remaining six samples needed for our population genomics analyses (Mexico/JAL-LaDEER-145365, 145340, 139093, E39931, 133706, 147248/2021, GISAID EPI ISL_1360409, 1360408, 1360412, 1360411, 1360407, 1360410). Sample collection of the Jalisco sequences (frequency of 0.69%, 9/1330) occurred during January 2021.

### Phylogenomics reveals convergent evolution and spread of mutations of concern in Mexico: identification of VOI P.4 and B.1.1.222/B.1.519

To further identify potential mutations of concern and characterize their phylogenetic associations, as well as their co-occurrence and frequency at the clade level, we constructed an ad hoc database for Mexican genome sequences, called Mexstrain, which includes selected references to allow for the correct establishment of phylogenetic relationships in the Nextstrain platform. The Mexstrain version used for our analyses consists of 4242 genome sequences (1554 from Mexico), and includes our newly generated high-quality genome sequences from asymptomatic carriers (five) and symptomatic patients (30) from Central Mexico (Guanajuato, San Luis Potosi and Jalisco States). As such, Mexstrain provides the universe of currently available genomic data for the pre-vaccination stage (March 2020 to February 2021) of the Covid-19 pandemic in Mexico, allowing us to identify the presence of additional spike protein mutations of concern in two sequences or more, at positions 13 (S → I), 18 (L → F), 20 (T → I), 26 (P → S), 144 (144 → X), 152 (W → C), 190 (R → M), 417 (K → N), 452 (L → R), 477 (S → N), 614 (D → G), 677 (Q → H), 681 (P → R/H) and 1176 (V → F). These samples are distributed throughout different clades and/or emerging lineages (Table 1).

**Table 1.** Mutations of concern with multiple occurrences in Mexico during the pre-vaccination stage of the Covid-19 pandemic.

Protein and amino acid (region)*	Mutation	VOC/VOI with this mutation†	Clades/lineages in Mexico‡	No. of sequences (State)§
S: 13 (NTD)	S → I	20C/452R, B.1.427/9	20C/B.1.427/9	44 (BCN, NLE, JAL, CMX,
				QUE, BCS, ROO)
	S → G		**20B/B.1.1.222**	1 (CMX)
S: 18 (NTD)	L → F	20h/501Y.V2, B.1.351	20G/B.1.2	2 (GUA, NLE)
		20J/501Y.V3, P.1	20B/B.1.1.29	1 (CMX)
			20B/B.1.1	1 (AGU)
			**20B/B.1.1.222**	2 (CMX, MEX)
			20B/B.1.1.237	1 (NLE)
			20B/B.1	1 (OAX)
S: 20 (NTD)	T → I	20J/501Y.V3, P.1	20C/B.1.2	1 (NLE)
			20A/B.1.189	1 (BCN)
	T → N		**20B/B.1.1.222**	3 (NLE, COA, CMX)
			20B/B.1	1 (OAX)
S: 26 (NTD)	P → S	20J/501Y.V3, P.1	20C/B.1.429	6 (CMX, NLE, QUE)
			**20B/B.1**	1 (OAX)
S: 138 (NTD)	D → Y	20J/501Y.V3, P.1	20A/B.1.189	1 (CMX)
			20A/B.1	1 (BCN)
			20A/B.1.404	16 (BCN, AGU)
			20B/B.1.1.244	1 (NLE)
	D → H		**20B/B.1.1.222**	1 (COL)
S: 144 (NTD)	144 → X	20I/501Y.V1, B.1.1.7	20A/B.1.189	1 (NLE)
		20A/484K, B.1.525	**20B/B.1.1.222**	1 (CMX)
S: 152 (NTD)	W → C	20C/452R, B.1.427/9	20C/B.1.427/9	44 (BCN, NLE, JAL, CMX,QUE, BCS, ROO)
	W → R		20A/B.1.94	1 (HID)
			20A/B.1.243	1 (BCN)
	W → L		20A/B.1	2 (CMX)
			**20B/B.1.1.222**	2 (CMX)
S: 190 (NTD)	R → M	20J/501Y.V3, P.1	20B/P.4	3 (JAL)
	R→ S		20A/B.1.243	1 (BCN)
S: 215 (NTD)	D → G	20 h/501Y.V2, B.1.351	20C/B.1.429	1 (JAL)
	D → Y		20A/B.1	1 (OAX)
			**20B/B.1.1.222**	1 (CMX)
S: 417 (RBD)	K → N	20h/501Y.V2, B.1.351	**20B/B.1.1.222**	1 (CMX)
	K → T	20J/501Y.V3 or P.1		
	
S: 452 (RBD, RBM)	L → R	20C/452R, B.1.427/9	20C/B.1.427/9	44 (BCN, NLE, JAL, CMX,
		21A/154K, B.1.617.1		QUE, BCS, ROO)
		21A/478K, B.1.617.2	20A/B.1.189	1 (NLE)
			20A/B.1.232	9 (BCN)
S: 477 (RBD, RBM)	S → N	20C/452R, B.1.427/9	20A/B.1	3 (BCN, SLP, VER)
	S → I		20A/B.1.404	3 (AGU)
			**20B/B.1.1.222**	1 (CMX)
S: 478 (RBD, RBM)	T → K	20B/478 K.V1, B.1.1.222	20B/B.1.1.29	1 (OAX)
		21A/478K, B.1.617.2	20B/B.1.1.85	1 (CMX)
			**20B/B.1.1.222**	494 (majority of states)
S: 484 (RBD, RBM)	E → K	20h/501Y.V2, B.1.351	20A/B.1	1 (SLP)
		20J/501Y.V3, P.1	20A/B.1.396	1 (SLP)
		20B/484K, P.2	20B/P.4	9 (JAL)
		20A/484K, B.1.525		
		20C/484K, B.1.526		
	E → Q	21A/154K, B.1.617.1	20A/B.1.243	1 (OAX)
S: 501 (RBD, RBM)	N → Y	20J/501Y.V1, P.1	20J/B.1.1.28.1	1 (JAL)
		20I/501YV3, B.1.1.7	20I/B.1.1.7	6 (TAM, NLE)
S: 614 (S1)	D → G	20I/501Y.V1, B.1.1.7	20A and 20B	1547 (majority of states)
		20h/501Y.V2, B.1.351		
		20J/501Y.V3, P.1		
		20B/484K, P.2		
		20A/484K, B.1.525		
		20C/484K, B.1.526		
		21B/154K, B.1.617.1		
		21A/478K, B.1.617.2		
S: 655 (S1)	H → Y	20J/501Y.V3, P.1	20B/B.1	1 (OAX)
	H → R			
S: 677 (S1)	Q → H	20A/484K, B.1.525	20G/B.1.2	1 (NLE)
			20A/B.1.189	2 (CMX)
			20A/B.1.241	1 (CMX)
			20A/B.1	3 (COA, SLP)
			20A/B.1.232	3 (COA, BCS).
			20B/B.1.1.220	2 (AGS, SLP)
			20B/B.1.1	1 (CMX)
			20B/B.1.1.28	1 (CMX)
	Q → P		**20B/B.1.1.222**	3 (GRO, CMX)
			20B/B.1.1.85	5 (NLE, COL, BCN, COA)
			20G/B.1.2	2 (COA, QUE)
S: 681 (S1)	P → H	20I/501Y.V1, B.1.1.7	20G/B.1.2	2 (YUC, QUE)
			20A/B.1.189	2 (QUE, NLE)
			20A/B.1.243	11 (TAM, NLE, QUE, COA)
			20B/B.1.1.29	1 (CMX)
			20B/B.1.1.	6 (CMX, COA)
			20B/B.1.1.344	1 (SLP)
			20B/B.1.1.29	1 (OAX)
			20B/B.1.1.85	1 (CMX)
			**20B/B.1.1.222**	491 (majority of states)
	P → R	21A/154K, B.1.617.1	20A/B.1	10 (CMX, MOR, BCN)
		21A/478K, B.1.617.2		
S: 701 (S2)	A → S	20 h/501Y.V2, B.1.351	20A/B.1	1 (MIC)
	A → V		20A/B.1	3 (CMX, TAM, COA)
		
S: 732 (S2)	T → A	20B/478 K.V1, B.1.1.222	20B/B.1.1.29	1 (OAX)
			**20B/B.1.1.222**	670 (majority of states)
	T → S		20B/B.1.1.85	9 (QRO, CMX, QUE, COA, BCN, NLE, COL)
S: 1176 (S2)	V → F	20J/501Y.V3, P.1	20B/B.1.1.28	2 (CMX, NLE)
		20B/484K, P.2	20B/P.4	9 (JAL)
N: 194 (IDR)	S → L	20@/B.1.1.289 (Mink)	20A/B.1||	342 (majority of states)
		20A/B.1.36		

*Mutations of concern in the spike (S) and nucleocapsid (N) proteins are based on previous reports (Plante *et al*., 2021) and in the co-variant data integrated by the Institute of Social and Preventive Medicine, University of Bern, Switzerland (Emma B. Hodcroft. 2021. ‘CoVariants: SARS-CoV-2 Mutations and Variants of Interest.’ https://covariants.org/). NTD, N-terminal domain (14–306). RBD, receptor binding domain (318–541). RBM, receptor binding motif (441–510). S1 (307–682) and S2 (683–1146) subunits. IDR, intrinsically disordered region. Only mutations occurring in more than one genome sequence during the period of study are reported.

†Known VOI or VOC that have been recorded in Mexico during the pre-vaccination stage are shown underlined, as these are part of the currently designated Covid-19 constellations (https://cov-lineages.org/constellations). VOI P.4 or B.1.1.28.4 is a sub-lineage closely related to the B.1.1.28.1 (P.1), B.1.1.28.2 (P.2) and B.1.1.28.3 (P.3) lineages.

‡Co-occurrence of mutations with T478K within 20B/B.1.1.222 or any other emerging lineage is shown in bold type. Refer to text for details on co-mutations, available in Table S3.

§Abbreviation of Mexican States (location) as recorded in GISAID, as follows (alphabetical order): AGS or AGU, Aguascalientes. BCN, Baja California. BCS, Baja California Sur. CMX, Mexico City. COA, Coahuila. COL, Colima. GUA, Guanajuato. GRO, Guerrero. HID, Hidalgo. JAL, Jalisco. MEX, Estado de Mexico. MIC, Michoacan. MOR, Morelos. NLE, Nuevo Leon. OAX, Oaxaca. QUE, Queretaro. QRO, Quintana Roo. SLP, San Luis Potosi. TAM, Tamaulipas. YUC, Yucatan. VER, Veracruz. The majority of S tates is used for 17 (out of 32) or more States.

||See Fig. 4 for a phylogenomic analysis of sub-clades 20A and lineages bearing the mutation N:S194L associated with the development of symptoms. Fig. S4 provides a phylogenomic analysis of all mutations included in Table 1.

More specifically, the genome data generated by Mexican authorities and other laboratories include sequences with the mutation E484K in the context of known VOC or VOI. This include: (i) a single sequence associated with 20J/501Y.V3 or P.1 (Mexico/JAL-InDRE_245/2021, GISAID EPI ISL 1008714); (ii) as mentioned in a previous section, three sequences from Jalisco annotated as 20B/P.2 or B.1.1.28.2 variants (Mexico/JAL-InDRE_371, 372, 373/2021) first identified by us and sequenced in parallel together with a further six sequences also identified by us (Mexico/JAL-LaDEER-133706, 139093, 145340, 145365, 147248, E39931/2021); and (iii) two sequences from the State of San Luis Potosí (Mexico/SLP-InDRE_192/2020 and Mexico/SLP-InDRE_454/2021), which belong to Nextstrain clade 20A (different sub-lineages within the Pangolin lineage B.1). This observation identifies the sequences of scenario (iii) as closer to the recently identified E484K-containing lineage 20A/S.484K (B.1.525) [15] and B.1.243.1 [49], than to 20B/P.2 (B.1.1.28.2). Both B.1.525 and B.1.243.1 were first reported in the USA, North East States and Arizona, respectively. As discussed in the final subsection of the Results, early cases of lineage B.1.243.1 were present in Mexico during the pre-vaccination stage.

**Fig. 1.**
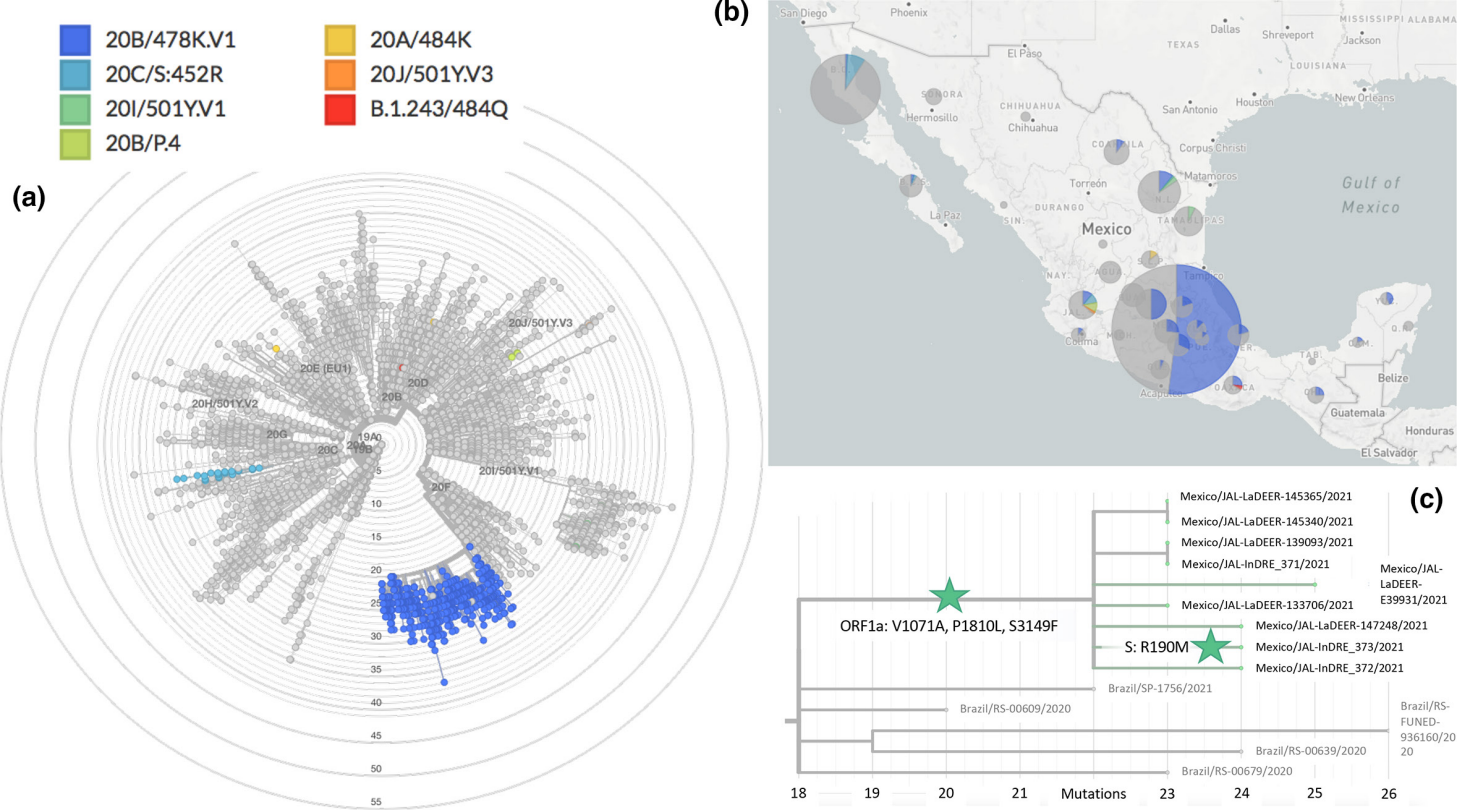
Phylogenomics of SARS-CoV-2 variants during the pre-vaccination stage in Mexico. (**a**) The Mexstrain phylogenomic tree showing VOI/VOC identified during the pre-vaccination stage. 20A/484K refers to the San Luis Potosi samples, belonging to lineages B.1 and B.1.319. (**b**) Geographical distribution of VOI/VOC in Mexico. (**c**) 20B/P.4 or 20B/B.1.1.28.4 evolving in Jalisco, Mexico, from P.2. ORF1a and spike protein amino acid mutations (ORF1a: V1071A, P1810L, S3149F; S: R190M) are indicated with green stars.

The above-mentioned scenarios (ii) and (iii), together with further E484K-containing variants identified by RT-qPCR screening (Fig. S2), places the States of San Luis Potosi and Jalisco as epicentres of E484K-containing SARS-CoV-2 VOI in Mexico. P.1 and P.2 share mutations E484K, D614G and V1176F (the last appearing to be specific to these closely related emerging lineages), but not N501Y. As expected from independently evolving VOC, mutation V1176F is absent from the two sequences from the State of San Luis Potosi. In contrast, all samples from Jalisco State, scenario (ii), are situated in the same lineage as 20B/P.2 or B.1.1.28.2. The sequence Mexico/JAL-InDRE_373/2021 includes the mutation R190M, an alternative version of mutation R190S present in the VOC P.1 or B.1.1.28.1, which may be signs of evolution in Mexico. Indeed, all Mexican sequences form a distinctive clade supported by the mutations ORF1a: V1071A, P1810L, S3149F (Figs 1c and S3). Based on these observations, and the fact that a similar situation has been reported in The Philippines involving the VOI P.3 (B.1.1.28.3, theta [50]), we propose to re-name the Jalisco P.2-like variant as P.4 or B.1.1.28.4. Given that P.3 has accumulated six further dangerous mutations in the spike protein, including E484K, N501Y and P681H, further investigation of P.4 in Mexico due to its evolutionary potential is recommended.

**Fig. 2.**
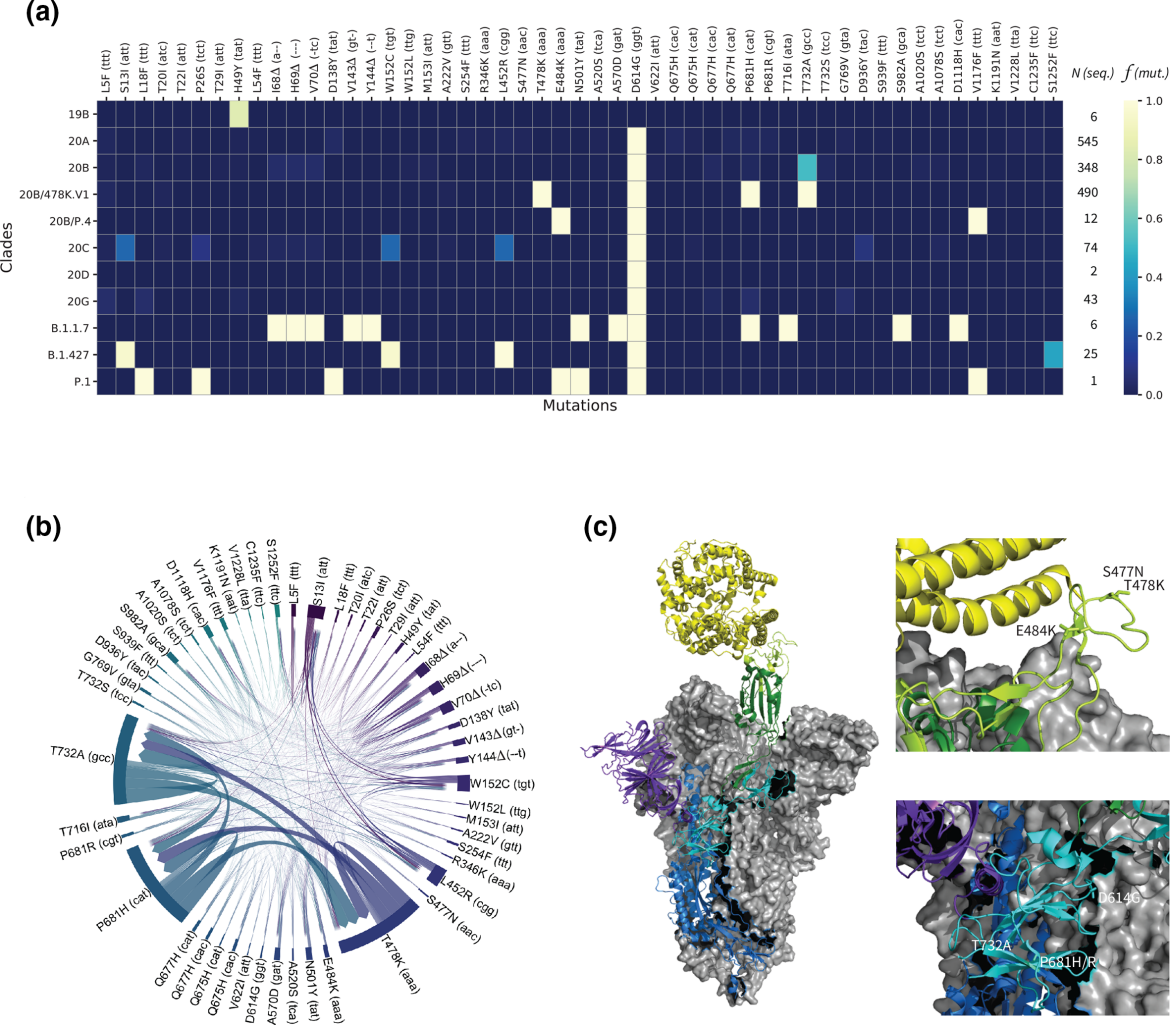
*S*ystematic sequence/structure analysis of the spike protein’s mutations of concern. (**a**) Heatmap highlighting relative frequencies *f* for 51 important mutations (out of 315) across 1552 viral genomes arranged in 11 clades (19B, 20A–D, 20B/478 K.V1, 20B/P.4, 20G, B.1.1.7, B.1.427, P.1). *N* indicates the number of available genome sequences per clade. See Fig. S5 for a heatmap including all mutations. (**b**) Chord diagram displaying the covariances between the 51 most frequent mutations. The thickness of the arrows denotes the strength of a covariance. (**c**) Left: electron microscopy structure (PDB:7A94 ref) of the trimeric spike (S) glycoprotein of SARS-CoV-2 bound to one angiotensin-converting ewnzyme-2 (ACE2, yellow). Two chains of the viral protein are shown in grey surfaces. The third chain comprises the N-terminal domain (purple), the S1 (cyan) and S2 (blue) protein subdomains, the receptor-binding domain (RBD, green), and the receptor-binding motif (RBM, light green). Right: positions of the important mutations S477N, T478K, E484K, D614G, P681H/R and T732A in two different regions.

Our phylogenomic analyses also revealed a different 484 mutation involving another amino acid substitution, E484Q, in the sequence Mexico/OAX-InDRE-61/2020 (August 2020, GISAID EPI ISL 576264). This sequence is shown in Fig. 1(a) and it corresponds to lineage B.1.243, further discussed below in the population genomics subsection. Glutamine (Q) is a basic residue, and therefore it is tempting to speculate that a similar effect to that generated by the basic residue lysine (K) may be in place, as proposed for the recently designated VOI 21A/S:154K evolving in India (B.1.617.1, kappa [51]). Irrespective of the codon usage of SARS-CoV-2 [52], the mutations from E to K (G → A) and from E to Q (G → C) involve single and non-sequential nucleotide point mutations, and thus they may offer alternative solutions to the same phenotype. A similar scenario with multiple combinations at the codon level, within different lineages in Mexico, can be seen in the mutation of concern S477N/I [53]. These mutations appear in seven sequences from different States distributed throughout different lineages (B.1.404 and B.1, for instance), and they involve mutations G → A and G → U in the second letter, respectively, which can be generated from two different sets of codons. The case of mutation 681 from P to R/H, present in the Mexican VOI 20B/478 K.V1 discussed next, also provides an important example of the SARS-CoV-2 genomic plasticity, concomitant with convergent evolution of mutations in the spike protein [54]. These cases, and others included in Table 1, Fig. S4, highlighted the importance of the characterization of allelic variation *de novo* as we do in the final population genomics subsection.

Mutagenesis of concern does not occur solely within the RBD of the spike glycoprotein, but also in its N-terminal domain [55], which is rich in glycosylation sites [56]. For instance, mutation L18F is a mutation of concern as it occurs in variants 20h/501Y.V2 or B.1.351 and 20J/501Y.V3 or P.1 and has been implicated in antibody scape. Within the period of analysis, this mutation occurs in Mexico in two forms: (i) in the single case associated with 20J/501Y.V3 or P.1 previously mentioned; and (ii) in two different lineages within several clades, accounting for at least six different backgrounds in a total of eight samples. A similar scenario for the NTD deletion 144X can be found in four samples from the North East of Mexico, Nuevo Leon and Tamaulipas States, associated with 20I/501Y.V1 or B.1.1.7, and more importantly, in seven samples identified in two different lineages, 20A/B.1.189 and 20B/B.1.1.222. These examples of convergent evolution of mutations of concern, together with many others, are also included in Table 1, Fig. S4.

In agreement with public announcements of Mexican Health authorities, a genomic lineage belonging to 20B/B.1.1.222, eventually leading into 20B/B.1.1.519, was confirmed simultaneously to the cognate report [57]. Worryingly, these lineages have in common between them – and with the recently designated VOC 21A/S:478K (B.1.617.2, delta [51], first identified in India and shown to undergo immune escape – mutations T478K and P681H/R. In addition, the Mexican variant shows mutation T732A. More than one-third of available sequences with a nationwide distribution with these mutations could be identified (Figs 1, 2 and S4). Although occurrence in Mexico City seems higher, the earliest sequence with these three mutations (annotated as lineage B.1.1.29) is from the South East State of Oaxaca, recorded as early as July 2020 (Mexico/OAX-InDRE_258/2020, GISAID EPI ISL 1054990).

We then systematically investigated overall mutation frequency and co-occurrence in lineages B.1.1.222 and B.1.1.519. For this, we first identified 315 mutations of concern in the spike protein that occur in at least one genome sequence during the period of analysis. We then analysed their incidences and their covariances across the 1552 genome sequences in which these mutations occur. The results of this analysis are presented in Figs 2(a, b) and S5. Fig. 2(a) highlights the incidences of the 51 most frequent mutations of concern within the 11 clades used to organize the data. We then selected the 51 mutations that were identified in at least five genome sequences overall, indicating the number of available genome sequences (*N*) per clade and the incidence of each mutation expressed as its relative frequency (*f*), also per clade. The highest incidences are depicted in yellow (close to 1.0), while low relative frequencies or nonexistent mutations are shown in shades of blue.

These analyses show that roughly 80% of the clade 19B sequences display the spike protein mutation H49Y, which appears to be unique to that clade. The insertions or deletions of the other mutations of concern illustrate the evolutionary relationships between subsequent clades, starting with clade 20A. Besides the conserved D614G mutation, sequences from the newly detected and proposed VOI 20B/478 K.V1 and 20B/P.4, share, in different combinations, mutations T478K, E484K, P681H/R with VOC alpha, gamma and delta (20I/B.1.1.7, 20J/P.1 and 21A/B.1.617.2). Variant 20B/478K.V1 has in addition the mutation T732A. Fig. 2(b) further validates the co-occurring mutations of concern mentioned above, particularly between T478K, P681H and T732A, from the dominant clade 20B of variant 20B/478K.V1. With only 25 sequences available, this analysis also identified covariances between S13I, L452R and W152C of the VOI B.1.427/9.

Among these results, although not at a high frequency, cases of T478K co-occurring with other spike protein mutations of concern could be detected, including: (i) K417N, which is present in 20h/501Y.V2 or B.1.351, identified in one sample (February 2021, GISAID EPI ISL 1137473); (ii) L18F, which is present in 20J/501Y.V3 or P.1, identified in one sequence from the State of Oaxaca, in the parental lineage B.1 (January 2021, GISAID EPI ISL 1168605); and (iii) the deletion of an amino acid in position 144, which occurs in 20I/501Y.V1 or B.1.1.7 and 20A/S.484K or B.1.525, in one sample from Mexico City (February 2020, GISAID EPI ISL 1181713). Similar to the convergent evolution of E484K leading to the potential VOI from the State of San Luis Potosi, mutation T478K is not only present in the entire country, but the sequence Mexico/OAX-InDRE_535/2021 (January 2021, GISAID EPI ISL 1168605) has it, and belongs to 20B/B.1. It is also interesting to note that mutations T478I/R, previously identified as dangerous [6, 58], could not be found in Mexico during the period of analysis.

### 3D structural analysis of mutations of concern occurring in Mexican VOI P.4 and B.1.1.222/B.1.1.519.

To predict the alleged roles the detected mutations of concern may take in the transmission and/or viral replication, we localized their positions onto the glycoprotein spike of SARS-CoV-2. For this, we focused on S477N, E484K and D614G as previously identified mutations of concern; and in mutations T478K, P681H/R and T732A present in the proposed VOI 478 K.V1. We used the electron microscopy trimeric structure (PDB:7A94) published by Benton and co-workers [48]. These authors reported several structures free or bound to one or several ACE2, of which we picked the example bound to a single ACE2 for simplicity, as shown in Fig. 2(c). In that figure, two of the three chains within the glycoprotein S are depicted in grey structures to keep our attention to the single chain interacting with ACE2 (yellow). The third chain includes the N-terminal domain (purple), the S1 (cyan) and S2 (blue) protein subdomains, the RBD (green), and the RBM (light green). Three mutations of concern, S477N, T478K and E484K, are in direct vicinity with the human receptor ACE2, located within the RBM. The mutations D614G and P681H/R are found in the S1 protein subdomain near T732A, taking part in S2. Overall, these structural projections emphasize the expected risks associated with these mutations, in particular for the proposed VOI 478 K.V1 emerging in Mexico.

Our reference-based (NC_045512.2) approach yielded high-quality read alignments for 85 of the 102 sequence libraries with a median value of uniquely mapped reads of 177 688 and first and third quartiles of 16 292 and 1751201, respectively (Table S3). These alignments were distributed in 44 libraries from Guanajuato, 32 from San Luis Potosí and nine from Jalisco. Once polymorphic sites were filtered by sequencing depth, quality of the alignments and population frequency, 83 (97 %) of the 85 initial samples were retained. From a total of 500 polymorphic sites across these samples, 59 high-quality SNVs/indels were retained, spanning the ~30 kb of the SARS-CoV-2 reference genome. These polymorphic sites include 53 SNVs and six indels ranging from 2 nt up to 7 nt in length. This result shows that the use of raw reads from multiple genomes increased the predictive power of genomic variation, more so for low-frequency variants that could be overseen in lower sample size experiments [59]

### Population genomics of SARS-CoV-2 in Central Mexico

**Fig. 3.**
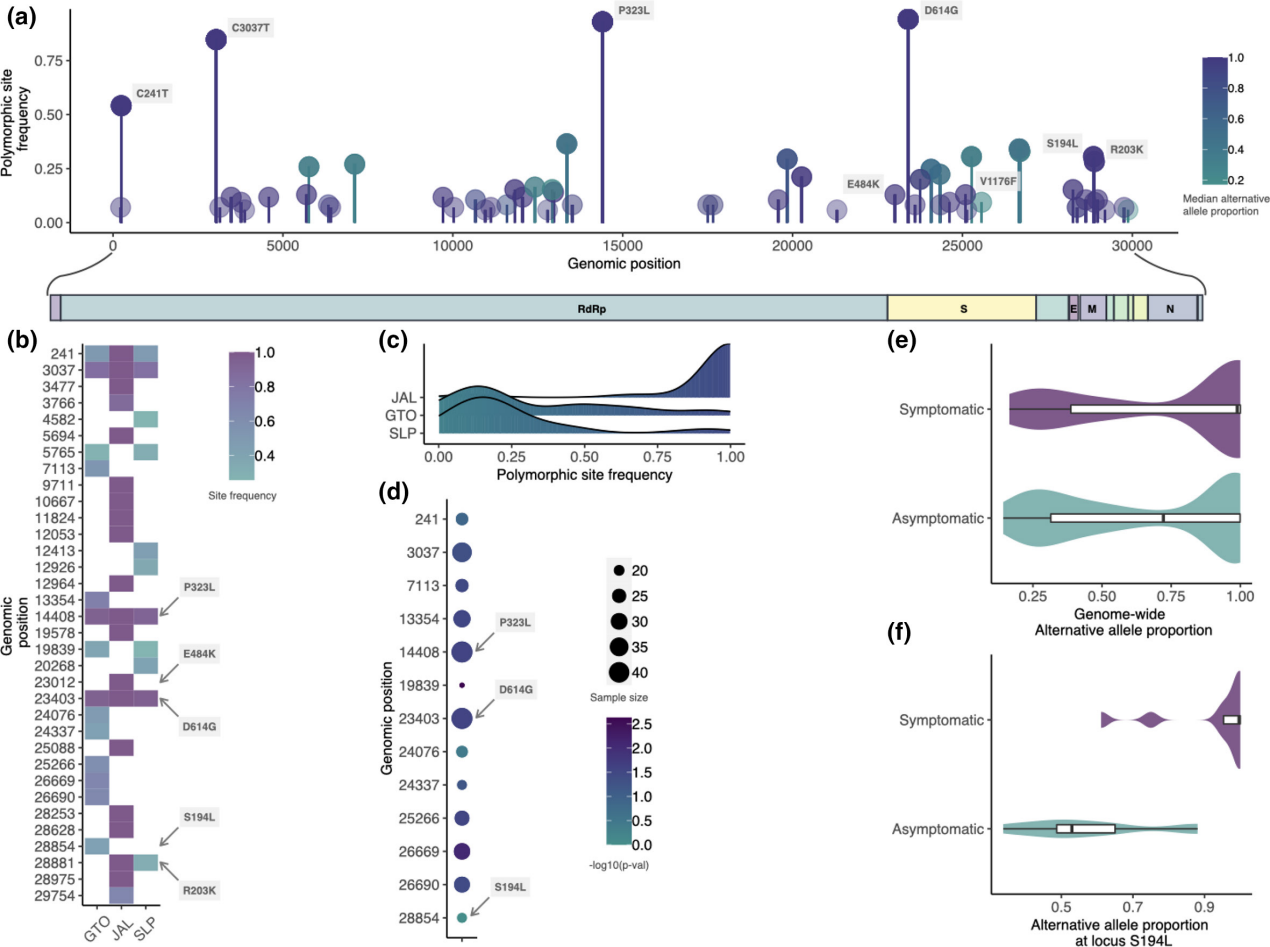
Landscape of genomic variation in central Mexico. (**a**) The genome-wide population frequency of SNVs plotted as lollipops. Colour reflects the per-site median allelic imbalance. (**b**) Heatmap showing the relative polymorphic site frequencies of the most common variants in the population on a per-locality manner basis. (**c**) Density plots showing the site frequency spectrum of the 59 high-quality polymorphic sites for each locality. (**d**) The discrete statistical association between polymorphic sites and the presence of symptoms shown as a dotplot. The size of the dots depicts the number of patients assessed while the colour denotes the −log_10_ of the *P*-values. Violin plots depict the distributions of the (e) genome-wide and (f) locus-specific (S194L) allelic imbalance for symptomatic and asymptomatic hosts within Guanajuato.

Our samples contained SNVs in one of the early strain types (VI) that spread out of China (C241T, C3037T, C14408T and A23403G mutations) which has the haplotype of allelic associations 241T-3037T-14408T-23403G, detected in high frequency worldwide (December 2020 [1]), and that may be involved in increasing the fitness of the SARS-CoV-2 virus [25]. Overall, we found that most of the genomic variants (SNVs+indels) occur in low frequencies, except for a few point variants, such as P323L in the RdRp protein, E484K, D614G and V1176F spanning the spike protein region, and R203K and S194L in the nucleocapsid region (Fig. 3a, b). Importantly, we found the mutation T478K in four of the 31 San Luis Potosi genomes, but it was filtered out of the population genetic analyses due to its low population frequency (<5%). The skewness of the site frequency spectrum towards rare polymorphisms (Fig. 3c) could be explained by neutral mutations linked to a beneficial mutation, such as D614G, increasing their frequencies due to hitchhiking (i.e. selective sweeps [60] Nielsen 2005). The prevalence of high-frequency polymorphisms in the JAL samples could be merely due to sampling bias, as the patients were pre-screened for the presence of the E484K mutation before the sequencing experiment, and it is the locality with the smallest sample size.

To evaluate the extent of substructure in our sampling, we used the 83 samples with high-quality polymorphic sites to generate a genetic covariance matrix representing the relatedness among samples, which in turn was used to perform a PCA. Principal components PC1, PC2 and PC3 explained 52, 21 and 15% of the variance in genetic differences, respectively. PC1 suggests overall admixture among the three localities except for a small portion of the samples from Guanajuato separated from those from San Luis Potosi and Jalisco (Fig. S6a).

By evaluating the allelic imbalance, as a quantitative measurement of the co-occurrence of variants intra-hosts, we found that for most polymorphic sites, the derived allele is found in higher proportions (Fig. S6b), mostly in Jalisco, which is probably explained by pre-screening of the E484K in this locality. In some polymorphic sites that were extracted from non-screened genomes, namely in Guanajuato, the ancestral allele dominates the sequencing reads in the sample.

The Guanajuato samples include asymptomatic patients, which could help explain the smaller cluster that we found (Fig. S6a). To investigate the discrete association between sequence polymorphism and the expression of symptoms, an exact binomial test was performed on 13 sites with at least 15 samples. Nine of these 13 sites showed a substantially higher proportion of asymptomatic patients compared to their symptomatic relatives (*P*<0.05), suggesting that most of the derived mutations could be associated with a mild or null expression of the disease (Fig. 3d), at least in this population genetic background. To further investigate the role of viral genetic composition on the presence or absence of symptoms in the Guanajuato cluster, we compared the median values of the allelic imbalance between symptomatic patients and asymptomatic carriers. We found that symptomatic patients have higher proportions of the derived allele (Wilcoxon test; effect size=0.12, *T*=17 808, *P*=0.00692) (Fig. 3e). By contrasting the allelic imbalance between symptomatic and asymptomatic hosts in a polymorphic site fashion, we found a single significant association of the C28854T mutation (ANOVA; *F*=20.5; *P*=0.003) (Fig. 3f). This SNV corresponds to the amino acid serine to leucine S194L, in the nucleocapsid region and has a population frequency of 30 % in Guanajuato. The probability that this is due to a founder effect is low, as Guanajuato genomes are not phylogenetically clustered (Fig. S7).

**Fig. 4.**
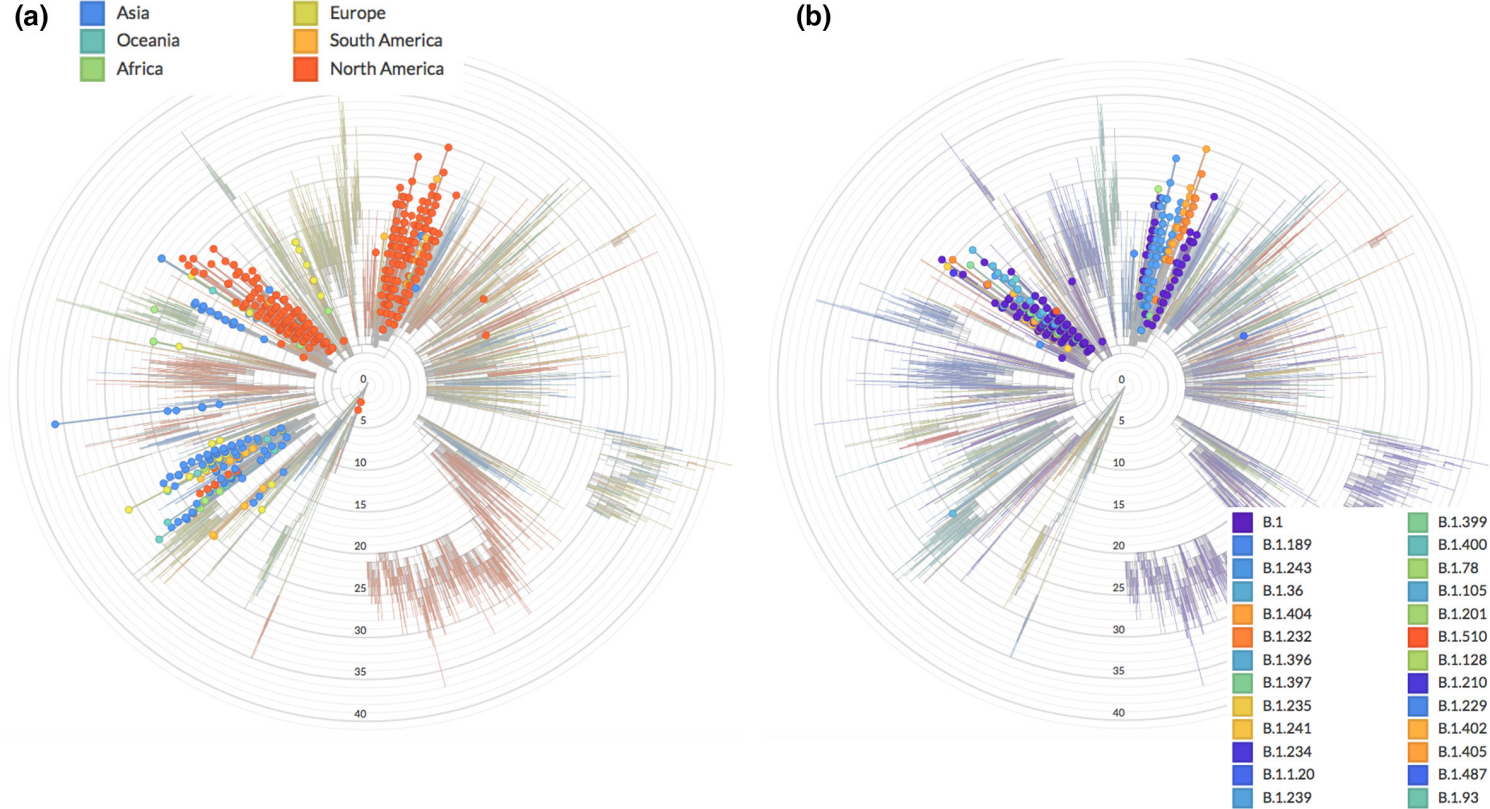
Presence of N:S194L in SARS-Cov-2 clades. (**a**) Worldwide occurrence of the N:S194L mutation using NextStrain. (**b**) Same analysis highlighting only sequences from Mexico distributed in two sub-clades consisting of a broad diversity of Pangolin lineages, dominated by B.1 but also showing B.1.243 with the potential to evolve the E484K mutation.

Motivated by these results, we analysed the prevalence of mutation N:S194L in the extended Nextstrain phylogeny, and found three major sub-clades within the 20A clade with high prevalence: (i) two closely related sub-clades dominated by the B.1 lineage, where most of the Mexican genomes are placed, including more than one-fifth of all sequences available for the period of analysis (342 out of 1554); and (ii) a mainly Asian sub-clade that includes the emerging lineage B.1.36.# (where # refers to many sub-lineages, e.g. 6, 10, 16, 18, 19, 21, 22, 25, 26 and 27) (Fig. 4a). However, many different Pangolin lineages are annotated within the implicated N:S194L-containing 20A sub-clade of scenario (i) (Fig. 4b). In addition to the B.1 lineage within these two 20A sub-clades, lineage B.1.243, with 78 genome sequences sharing the point mutation N:S194L and the deletion NS9c:Q41* (22.8 % of total sequences within these two 20A clades and 5.1 % of the total genome sequences), were of note. Analogous to the E484K mutation present in the parental lineage B.1.36 of scenario (ii), which has been implicated in regional outbreaks in India [61], the proposed new Mexican lineage of scenario (i) includes the two San Luis Potosi E484K-containing variants identified after RT-qPCR screening (lineages B.1 and B.1.319), and the Oaxaca E484Q variant appearing early on during the pandemic, which corresponds to lineage B.1.243. The potential for evolving the dangerous E484K/Q mutation within this Mexican lineage is supported by the recent detection of the sub-lineage B.1.243.1 in Arizona, USA, which includes mutation E484K [49]. Together, these observations warrant further investigation and direct surveillance as potentially dangerous nucleocapsid *and* spike protein mutations accumulate during the vaccination stage.

## Discussion

At the moment of emergence of all SARS-CoV-2 mutations leading to variants and constellations, a massive or global immune pressure elicited by vaccination or antiviral treatments was not present. Indeed, it has been reported that reinfections of SARS-CoV-2 can occur [62–65]. Rapid and independent viral evolution has been observed in other pathogens such as the respiratory syncytial virus (RSV). In 2009, during the influenza A/H1N1 pandemic, a 72-nt duplication in the G gene of RSV-A occurred. During the following 3 years, multiple independent duplication events occurred around the world [66]. In fact, convergent evolution of the gene G in RSV-A and RSV-B have been reported [67]. The mechanism that drives RSV and SARS-CoV-2 convergent evolution may be similar. This may involve the lack of a specific antiviral treatment, the lack of vaccines (SARS-CoV-2 convergent events occurred before the distribution of vaccines), a similar R0 of the two pathogens, a global high transmission rate and the capacity to escape from the humoral immune response.

During the pre-vaccination stage in Mexico, our analyses revealed two potential VOI with mutations of concern in the spike protein: 20B/478K.V1 or B.1.1.222/B0.1.1.519 and P.4 or B.1.1.28.4, plus an evolutionary event with the potential to lead to a dangerous B.1.243 sub-lineage. On the one hand, although further data are needed to confirm the E484K-containing sequences detected in San Luis Potosi as VOI, which occurs within a clade dominated by the B.1.243 lineage, our results emphasize the potential of a combined targeted RT-qPCR screening and genome sequencing approach to anticipate epidemiological hotspots. It is tempting to speculate that these sequences could be related to the origin of the E484K-containing variant B.1.243.1 detected in Arizona [49], a southern state in the USA that borders the Mexican northern states of Sonora and Chihuahua. However, further data are required to assess the phylodynamics of this lineage and distinguish between common origin or convergent evolution. In the meantime, the San Luis Potosi E484K-containing variants are different from the closely related and previously reported P.1, P.2 and, recently, P.3 sequences [50]. Although the proposed P.4 variant still appeared at a low frequency (0.68%) during the period of analysis, it can be concluded that this variant is the result of an early P.2 entry followed by local evolution (including mutation of R190 into M instead of S). These results are in line with data suggesting convergent evolution and global spread of the virus containing the E484K mutation during the pre-vaccination stage [14, 15, 54, 68]. Moreover, during revision of this manuscript, the proposed VOI P.4 showed a significant increase of its prevalence, mainly in the state of Jalisco (data not shown).

On the other hand, 20B/478K.V1, with mutations T478K, P681R/H and T732A in its spike protein, can undoubtedly be classified as a VOI, with the potential to become a VOC. This has been suggested as well by other authors [15, 57, 69], who simultaneously reported the occurrence of this variant in Mexico, USA and Europe. Importantly, during the pre-vaccination stage, this VOI was ascribed as the B.1.1.222 Mexico and USA lineage, which at the time of submission appeared more often as B.1.1.519. Beyond nomenclature, the phenotypic implications of this VOI, including potential clinical outcomes and impact upon diagnostics, represent a pressing issue that remains to be addressed. Of particular concern are the worrisome Covid-19 circumstances in India, which may relate to the recently demonstrated ability of the rapidly emerging VOC 21A/B.1.617.2 to escape the immune system [51]. Like variant 20A/478K.V1, the latter VOC includes the mutations T478K and P681H/R. Indeed, our 3D structural analysis of the spike protein hints towards two regional hotspots for viral mutagenesis. The first one is in the flexible loop of the RBD, and includes S477N, T478K and E484K. These mutations may affect the interactions between the spike protein and the ACE2 receptor [53, 58]. The other hotspot is within S1–S2 subdomains, and which includes the furin-like protease cleavage site [70, 71], and contains D614G, P681H/R and T732A. Mutations T478K and S477N, involving changes from similar hydroxylated side-chains (T and S) into positively charged basic amino acids (K and N), may have analogous functional roles, reinforcing the ability of the virus to bind to the human ACE2 receptor [70]. Interestingly, S477 has been identified as the most flexible amino acid within the RBM and with the largest number of mutations [53], which might be stabilized by mutation T478K. A case exemplifying a similar scenario in which physically close mutations contribute towards an analogous structural solution is provided by the spike protein mutation Q613H. In this case, mutation Q613H defining the emerging lineage A.23.1, from Uganda, may have taken the role of the mutation D614G in B lineages during evolution of A lineages [72].

Our population-level results are consistent with a previous report that highlights the dual role of spike and nucleocapsid proteins in adaptive evolution of SARS-CoV-2 to their hosts [26]. The discrete association of both D614G and P323L to an asymptomatic phenotype could mean that those fixed mutations enhance transmissibility by bypassing an immune response [1, 73]. So, it is possible that those mutations could produce an asymptomatic phenotype in carriers with a Mexican genetic background. Also, as far as we are aware, the association of P323L to severity disease has been only tested in symptomatic patients [74] so ours is the first observation of P323L being associated with an asymptomatic phenotype. P323L affects the function of the RNA-dependent RNA polymerase, associated with an increase in mutation rate. A hypothesis – yet to be tested – is that mutations P323L–D614G reinforce the asymptomatic phenotype, at least in a Mexican genetic background.

Our finding that symptomatic hosts are associated with a higher proportion of the N:S194L-derived allele relative to the Wuhan allele is particularly intriguing. The SARS-CoV-2 nucleocapsid protein N is a multidomain RNA-binding protein that is found inside the viral envelope and it is required by the virus to maintain its structure and viability once the virus has entered the cell [75, 76]. The mutation we identified, N:S194L, is within the central domain composed of intrinsically disordered regions (IDRs) [75], which are conformationally flexible and promiscuous [77], and are increasingly recognized as important in increased viral transmission [76, 78]. Mutations in positions 193, 197, 203 and 204 of the linker IDR have been mapped to the loops connecting disordered and structured regions [76], implicating these sites in increased transmission. Interestingly, variants of lineage B.1.36.# with mutation N:S194L have been associated with higher mortality in Gujarat, India [79]. Joshi *et al.* found in March 2021 that this variant had an allele frequency of 47.62 and 7.25 % in deceased patients, compared to 35.16 and 3.20 % of the same variant in recovered patients, from the Gujarat and global datasets, respectively (although the difference was only significant in the global dataset). Additionally, the N:S194L mutation also occurs at a very high frequency (89.9%, *N*=1270) in lineage B.1.1.289 ([80]), isolated in Denmark and shown to co-infect humans and minks [21]. The evolutionary and functional relationships between the Mexican sub-clades bearing this mutation, including lineage B.1.243 (but also other lineages, such as, B.1 and B.1.189), and the previously reported and partially characterized lineages B.1.36.# and B.1.1.289, remain to be investigated.

A clear advantage of the population-level approach adopted here is that it allows us to identify and characterize SNVs from fragmented genomes. This calls for an additional global repository that can be used for population-level studies. As was previously proposed by Yang *et al*. [25], SNVs may become an important consideration in SARS-CoV-2 classification, surveillance and tracking as viruses travel through the world and accumulate additional mutations by convergent evolution. Our finding of the mutation N:S194L contributes to an additional site for RT-qPCR screening of samples outside of the spike region. Future population genomic and phylogenomic studies of SARS-CoV-2 must consider the protein multidimensional landscape of the virus, both spike and nucleocapsid. Understanding the role of intra-host selection pressures and their subsequent transmission as vaccination continues is critical. For instance, we do not know if new variants occur mostly as the result of admixture, or the mixtures of different variants within the same person, nor their relationship to various vaccines or mixed vaccination in a diverse human background. As different vaccines are used within and across regions, and varying timing of vaccination programmes across countries takes place, genome surveillance will still be of essence to monitor and identify emerging phylogenetic lineages that could become VOCs. Foremost, the recent appearance of various sub-strains that result from prolonged individual infections and subsequent transmission, as well as regional surges and episodic outbreaks [59] prompt more phylogenomic studies coupled with population-level analyses that can help provide detailed recommendations to decision-makers.

## Supplementary Data

Supplementary material 1

Supplementary material 2
